# Meropenem-Resistant Pandoraea Pneumonia in a Critically Ill Patient With COVID-19

**DOI:** 10.7759/cureus.19498

**Published:** 2021-11-12

**Authors:** Mohammad M Dlewati, Pyi Phyo Aung, Kyeeun Park, Jose A Rodriguez, Kenneth K Poon

**Affiliations:** 1 Internal Medicine, Memorial Healthcare System, Hollywood, USA; 2 Infectious Disease, University of Florida, Gainesville, USA; 3 Infectious Disease, Memorial Healthcare System, Hollywood, USA

**Keywords:** proinflammatory, critical care, 16s ribosomal dna sequencing, covid-19, carbapenems, antibiotic resistance, ventilator associated pneumonia, pandoraea species

## Abstract

Among patients infected with respiratory viruses, primary coinfection or secondary bacterial pneumonia is common in the severely ill. *Pandoraea* are multi-drug resistant gram-negative bacilli that have been newly classified in the past 20 years. We present the first reported case of *Pandoraea* co-infection with SARS-CoV-2 infection.

A critically ill gentleman with COVID-19 in acute respiratory distress syndrome (ARDS) requiring mechanical ventilation developed ventilator-associated bacterial pneumonia (VAP). Initial sputum cultures grew *Pandoraea* species, with subsequent cultures growing *P. aeruginosa*, *and K. pneumoniae* as well. The patient failed to improve despite several antibiotic regimens including meropenem. Send-out reference laboratory testing of the *Pandoraea* species showed susceptibility to amikacin, ciprofloxacin, levofloxacin, imipenem, and minocycline, but resistance to aztreonam, cefepime, ceftazidime, and meropenem. The patient had deteriorated to multi-organ failure by the time minocycline was initiated, and his family had transitioned him into hospice care.

Carbapenems are vital agents in the treatment of VAP. *Pandoraea *species are often resistant to meropenem but often retain in-vitro sensitivity to imipenem-cilastin. Although mainly isolated from respiratory specimens of patients with cystic fibrosis, cases of infection in non-cystic fibrosis patients have been increasingly recognized. The presentation of this case aims to increase awareness of the high drug resistance of this rising species and reduce delays in treatment, especially in COVID-19 coinfection.

## Introduction

Among patients infected with respiratory viruses, primary coinfection or secondary bacterial pneumonia is common in the severely ill [1]. This phenomenon also exists with COVID-19, and the coinfection rate of severe patients is significantly higher than that of non-severe patients. In a systematic review and meta-analysis of thirty studies including 3834 patients with COVID-19, the most common bacteria responsible for co-infections were *Mycoplasma pneumonia*, *Pseudomonas aeruginosa*, and *Haemophilus influenza *[2-3]. Ventilator-associated pneumonia (VAP) is a common complication in mechanically ventilated COVID-19 patients. Superimposed bacterial infections have the potential to worsen a patient’s clinical condition and increase mortality, as well as prolong and increase the costs of hospitalization. Preventing, identifying, and treating early VAP can increase the chances of successful treatment in patients with COVID-19 [4].

*Pandoraea* species are gram-negative bacilli described in the past 20 years [5]. Although mainly isolated from respiratory specimens of patients with cystic fibrosis, cases of infection in non-cystic fibrosis patients have been increasingly recognized [6-8]. These species belong to a group of non-lactose fermenting gram-negative bacilli notorious for multi-drug resistance which includes *Pseudomonas aeruginosa, *the* Acinetobacter calcoaceticus-baumannii *complex*, Stenotrophomonas maltophilia,* and *Burkholderia cepacia* complex [9-10]. Clinically, identification and treatment can prove challenging as is illustrated in our case. To our knowledge, we present the first reported case of *Pandoraea *co-infection with SARS-CoV-2 infection. The presentation of this case aims to increase awareness of the high drug resistance of this rising species and reduce delays in treatment, especially in individuals with COVID-19. 

## Case presentation

A 69-year-old male presented to the emergency department with a chief complaint of progressively worsening dyspnea for one week since testing positive for SARS-COV-2 at a community screening site. His only other symptoms were cough and nasal congestion. He was afebrile, tachypneic, and hypoxemic with SpO_2_ of 86% on room air. Diffuse bilateral crackles were heard on auscultation of the chest, with an otherwise unremarkable physical exam. A chest X-ray (CXR) revealed bilateral peripherally concentrated lung infiltrates. Initial labs are presented in (Table 1) below. 

**Table 1:** Admission Labs FEU: Fibrinogen equivalent units

Lab	Value (units)	Reference values
Hemoglobin	16.6 (g/dL)	13.0-17.0 g/dL
White Blood Cell Count	9x10^3 ^(cells/uL)	3.5-10^3^ cells/uL
Absolute Lymphocyte Count	740 (cells/uL)	850-3000 cells/uL
D-dimer	0.67 (mg/L FEU)	0-0.49 mg/L FEU
C-reactive Protein	12.5 (mg/dL)	<0.30 mg/dL
Lactate Dehydrogenase	543 (units/L)	87-246 units/L
Ferritin	1980.2 (ng/mL)	22-322 ng/mL
Lactic Acid	2.0 (mmol/L)	0.4-2.0 mmol/L
SARS-COV-2 RT-PCR	positive	negative

**Figure 1:**
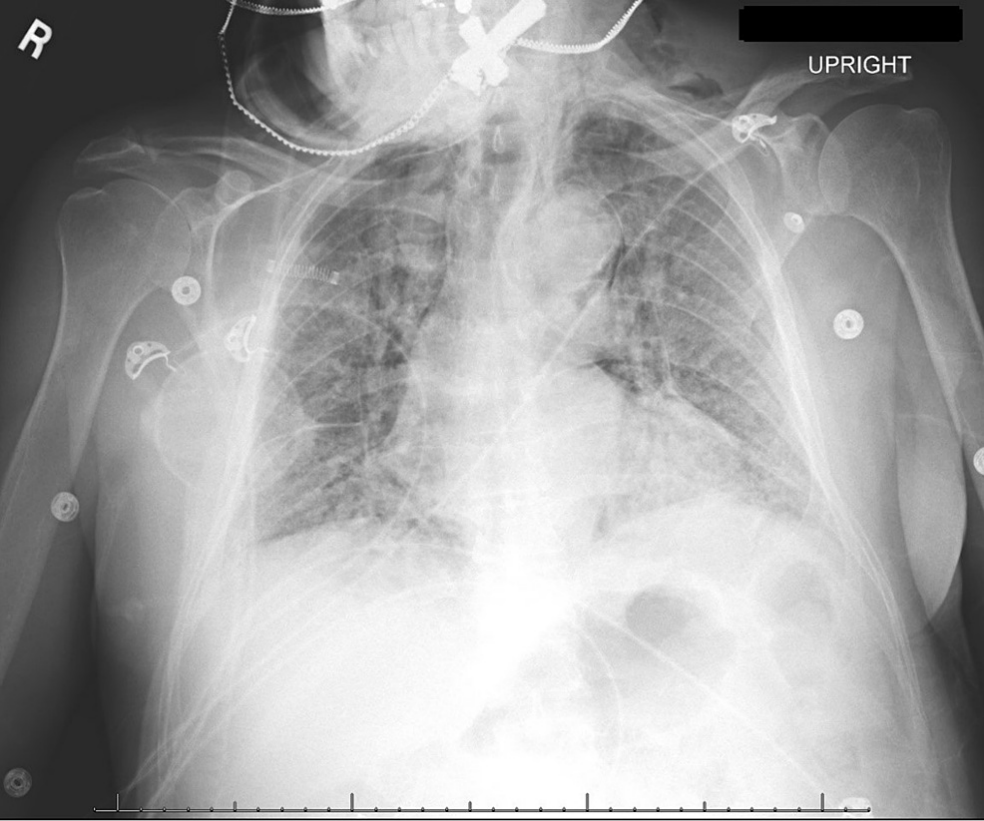
Chest X-ray day 10 of admission revealing extensive bilateral infiltrates with pneumomediastinum and subcutaneous emphysema.

Due to worsening oxygenation despite nasal cannula oxygen supplementation at 4 L/min, non-invasive bilevel positive airway pressure (BiPAP) ventilation was started via a face mask with 100% FiO2. He was admitted with enhanced respiratory precautions for further management. Initial management included empiric antibiotic coverage with ceftriaxone and doxycycline, dexamethasone, remdesivir, and prophylactic anticoagulation with enoxaparin. Over the course of the first few days of admission, the patient continued to require high levels of oxygen supplementation, alternating between BiPAP and non-rebreather mask (NRM) with FiO2 over 90%. On day 8 of admission, convalescent plasma became available and he was transfused one unit. On days 9 and 10 of admission, the patient had worsening hypoxemia and respiratory distress despite non-invasive respiratory support. A CXR revealed persistent bilateral infiltrates with a new pneumomediastinum and subcutaneous emphysema (Figure 1).

The WBC count had increased to 29.6 x 10³/μL. The patient was intubated, placed on mechanical ventilation, and started on cefepime empirically. Vasopressor support was temporarily required for hemodynamic support.

Blood and sputum cultures were drawn, however, no growth was found, and the patient's leukocytosis showed improvement to 13.2 x 10³/μL with the completion of a 7-day course of cefepime. The patient continued to be hypoxemic, and leukocytosis started to markedly increase once again. Repeat sputum cultures retrieved by endotracheal tube suction grew *Pandoraea* species with no available sensitivities. Hence, cefepime was reinitiated. A 5-day course of fluconazole was given to treat *Candida albicans *catheter-associated urinary tract infection. After 7 days, leukocytosis persisted, prompting reculture. Repeat sputum cultures heavily grew *Pandoraea *species, *Pseudomonas aeruginosa*, and *Klebsiella pneumoniae*. The latter two were sensitive to cefepime, but *Pandoraea *susceptibilities were still not available. A specimen was sent out to a reference laboratory for sensitivity testing. The leukocytosis and hypoxemia worsened despite cefepime treatment. Hence, antimicrobial therapy was escalated to meropenem and micafungin. After more than 26 days from the initial growth of the *Pandoraea *species, the reference laboratory report returned noting its resistance to meropenem and cefepime. At this time, the patient had already developed respiratory failure refractory to multiple ventilation strategies, multi-organ failure, disseminated intravascular coagulation, severe anemia due to diffuse hemorrhage, and the WBC was 30.3 x 10^3^/uL. Intravenous minocycline was added, and meropenem was continued. Two days later his WBC count improved to 13.7 x 10^3^/uL. However, at this point, the family's goals of care had changed, and the patient was transitioned to hospice care with the withdrawal of life support and subsequent expiration shortly thereafter.

## Discussion

SARS-CoV-2 virus was first identified in Wuhan, China in December 2019 and the disease caused by the virus has become universally recognized and described as coronavirus disease 2019 (COVID-19). On March 11, 2020, the World Health Organization (WHO) declared COVID-19 a pandemic, and to this day, the aggressive spread of this virus continues across the globe [9]. Therefore our understanding of the immunopathology of COVID-19 and its interaction with the human host under different pathologic conditions remains an area of significant interest. The lungs are the main organ involved in COVID-19; the virus infects the alveolar epithelial cells leading to dysfunction, progressive accumulation of debris and fluid in the lungs, and acute respiratory distress syndrome (ARDS). In severe cases of COVID-19, systemic immune over-activation leading to cytokine storm is a major factor for high mortality, multi-organ failure, ARDS, and disseminated intravascular coagulation. Mechanical ventilation is a common supportive treatment in these cases [11-12].

The pathogenicity of *Pandoraea *is primarily via proinflammatory responses secondary to the accumulation of cytokines such as IL-6 and IL-8. In a study of the in-vitro virulence characteristics of 17 *Pandoraea *isolates, only three isolates showed an ability to invade lung epithelial cells. However, all 17 isolates triggered up to a 19-fold elevation of IL-6 (two- to 19-fold) and 50-fold elevation of IL-8 compared to a control strain of Escherichia coli [13,14]. These proinflammatory responses are similarly found in the “cytokine storm” of COVID-19 [15]. Our patient had a marked leukocytosis, severely elevated inflammatory markers with a peak ferritin level of 4606 ng/ml, severe ARDS, and accelerated multi-organ dysfunction. These findings and the studies cited above suggest that *Pandoraea *may have amplified the already exaggerated inflammatory response associated with COVID-19. Comparing other potential cases of SARS-COV-2 in which *Pandoraea *is isolated will provide further insights into the immunopathology of *Pandoraea *species and its clinical significance.

It has been noted in the literature that accurate genus and species-level identification by routine clinical microbiology methods is difficult, and differentiation from the *Burkholderia cepacia *complex organisms may be especially problematic, making this an under-reported pathogen. Proper identification may require 16S ribosomal DNA sequencing or further molecular techniques [5,8,13,14]. In our case, a conventional automated system was able to correctly identify *Pandoraea *to the genus level, but did not deliver results for antibiotic susceptibility. The subsequent lag due to the technicalities of sending a specimen to a reference laboratory caused significant delays in obtaining vital data.

Our microbiology laboratory used the Biomerieux VITEK ® MS automated mass spectrometry microbial identification system and VITEK® 2 microbial ID/AST testing system to identify *Pandoraea *to the genus level. The specimen was sent to a reference laboratory for antibiotic susceptibility testing. Susceptibility testing was then performed by broth microdilution using custom-made MIC panels and interpreted according to CLSI guidelines. It was found to be resistant to aztreonam (MIC ug/mL>= 64), cefepime (MIC>=64), ceftazidime (MIC>=32). and meropenem (MIC>=16), Intermediate susceptibility to gentamicin (MIC 8), and susceptible to amikacin, ciprofloxacin, imipenem, levofloxacin, and minocycline.

Several mainstream commercial clinical references utilized at our institution had little information regarding “*Pandoraea*”. There was a 26 day interval from the first isolation of *Pandoraea *in the patient’s sputum, until therapy with an antibiotic with adequate susceptibility was initiated. The unique pattern of resistance to carbapenems conferred by carbapenem hydrolyzing oxacillinases (resistance to meropenem but susceptibility to imipenem-cilastin) has been recognized in the scarce literature [6,16-17]. As a nonfermenting gram-negative bacillus, both seem to be appropriate agents, especially in the presence of *Pseudomonas *coinfection. This is especially relevant in the realms of critical care where mainstay of ventilator associated pneumonia treatment includes carbapenems [18].

## Conclusions

In conclusion, *Pandoraea *species can be difficult to successfully treat in a timely fashion and are potentially life-threatening pathogens. There is a need for increased awareness about the specific antibiotic resistance pattern of *Pandoraea, *particularly with resistance to meropenem. The co-presence of *Pandoraea* with SARS-COV-2 may have caused a compounded inflammatory response. Thus, exploration of possible pro-inflammatory interactions with SARS-CoV-2 are warranted.

## References

[1] Klein EY, Monteforte B, Gupta A, Jiang W, May L, Hsieh YH, Dugas A (2016). The frequency of influenza and bacterial coinfection: a systematic review and meta-analysis. Influenza Other Respir Viruses.

[2] Lansbury L, Lim B, Baskaran V, Lim WS (2020). Co-infections in people with COVID-19: a systematic review and meta-analysis. J Infect.

[3] Dudoignon E, Caméléna F, Deniau B (2021). Bacterial pneumonia in COVID-19 critically ill patients: a case series. Clin Infect Dis.

[4] Póvoa HC, Chianca GC, Iorio NL (2020). COVID-19: an alert to ventilator-associated bacterial pneumonia. Infect Dis Ther.

[5] Coenye T, Liu L, Vandamme P, LiPuma JJ (2001). Identification of Pandoraea species by 16S ribosomal DNA-based PCR assays. J Clin Microbiol.

[6] Lin C, Luo N, Xu Q, Zhang J, Cai M, Zheng G, Yang P (2019). Pneumonia due to Pandoraea Apista after evacuation of traumatic intracranial hematomas:a case report and literature review. BMC Infect Dis.

[7] Gautam V, Singhal L, Ray P (2011). Burkholderia cepacia complex: beyond pseudomonas and acinetobacter. Indian J Med Microbiol.

[8] Pimentel JD, MacLeod C (2008). Misidentification of Pandoraea sputorum isolated from sputum of a patient with cystic fibrosis and review of Pandoraea species infections in transplant patients. J Clin Microbiol.

[9] Mehrad B, Clark NM, Zhanel GG, Lynch JP 3rd (2015). Antimicrobial resistance in hospital-acquired gram-negative bacterial infections. Chest.

[10] Segonds C, Paute S, Chabanon G (2003). Use of amplified ribosomal DNA restriction analysis for identification of Ralstonia and Pandoraea species: interest in determination of the respiratory bacterial flora in patients with cystic fibrosis. J Clin Microbiol.

[11] (2020). WHO. Coronavirus disease (COVID‐2019) situation reports. https://www.who.int/emergencies/diseases/novel-coronavirus-2019/situation-reports..

[12] Li X, Ma X (2020). Acute respiratory failure in COVID-19: is it "typical" ARDS?. Crit Care.

[13] Caraher E, Collins J, Herbert G (2008). Evaluation of in vitro virulence characteristics of the genus Pandoraea in lung epithelial cells. J Med Microbiol.

[14] Costello A, Herbert G, Fabunmi L (2011). Virulence of an emerging respiratory pathogen, genus Pandoraea, in vivo and its interactions with lung epithelial cells. J Med Microbiol.

[15] Song P, Li W, Xie J, Hou Y, You C (2020). Cytokine storm induced by SARS-CoV-2. Clin Chim Acta.

[16] Schneider I, Bauernfeind A (2015). Intrinsic carbapenem-hydrolyzing oxacillinases from members of the genus Pandoraea. Antimicrob Agents Chemother.

[17] Stryjewski ME, LiPuma JJ, Messier RH Jr, Reller LB, Alexander BD (2003). Sepsis, multiple organ failure, and death due to Pandoraea pnomenusa infection after lung transplantation. J Clin Microbiol.

[18] Garnacho-Montero J, Corcia-Palomo Y, Amaya-Villar R, Martin-Villen L (2014). How to treat VAP due to MDR pathogens in ICU patients. BMC Infect Dis.

